# A Rare Presentation of Asymptomatic Spontaneous Pneumomediastinum

**DOI:** 10.7759/cureus.13695

**Published:** 2021-03-04

**Authors:** Thomas Y Sun, Allison Ferrara

**Affiliations:** 1 Internal Medicine, University of Central Florida College of Medicine, Orlando, USA; 2 Internal Medicine, Osceola Regional Medical Center, Kissimmee, USA

**Keywords:** pneumomediastinum, asymptomatic pneumomediastinum

## Abstract

Spontaneous pneumomediastinum is a rare condition characterized by interstitial air within the mediastinum without any obvious causative factors. It is most commonly found in young men, and the clinical presentation is typically associated with chest or neck pain and dyspnea. Objective findings can include subcutaneous emphysema of the neck and chest. Asymptomatic cases are exceedingly rare. In this report, we present the case of a 20-year-old woman who presented with acute psychosis and the incidental imaging finding of pneumomediastinum without any associated clinical signs or symptoms.

## Introduction

Pneumomediastinum, or mediastinal emphysema, is a medical condition characterized by the presence of air in the mediastinum. Its pathophysiology starts with the escape of free alveolar air into the pulmonary interstitium. Negative pressures then pull the air along perivascular sheaths, eventually dissecting the hilum and spreading into the mediastinum. Risk factors include increased alveolar pressure, increased mediastinal pressure, or a dramatic decrease in intravascular pressure [1]. Cases may either be spontaneous or secondary to iatrogenic, traumatic, pulmonary, infectious, or drug-induced etiologies [2]. The clinical incidence has been reported to be between two to four per 100,000 cases, although some believe that the incidence is higher due to underdiagnosis [3,4,5]. Generally, pneumomediastinum occurs in younger patients and affects males about twice as much as females [6]. Clinically, the commonly reported symptoms include retrosternal chest pain (60-100% of cases), coughing spells (80%), dyspnea (75%), and neck pain (36%). On physical exam, the most common findings are subcutaneous emphysema (70%), rhinolalia (nasal speech tone), and hoarseness. The pathognomonic finding of pneumomediastinum is the Hamman’s sign, described as a crunching or clicking heard on cardiac auscultation. Interestingly, this is only observed in around 12% of cases [7]. Asymptomatic cases are exceedingly rare and seldom described in the literature [8,9,10].

## Case presentation

A 20-year-old woman with a history of polysubstance abuse presented to the emergency department (ED) with an episode of acute psychosis. She had been hospitalized for a similar episode one month earlier and diagnosed with drug-induced psychosis. As per the patient's friend, prior to that hospitalization, the patient had been vomiting and agitated with aggressive behavior for two hours. The friend also reported alcohol and illicit drug use, but specific details were not disclosed. Upon presentation to the ED, the patient had significant auditory and visual hallucinations. She denied any chest or neck pain, palpitations, coughing, or dyspnea. Her social history was significant for alcohol and illicit drug use, most significantly marijuana.

On initial evaluation, she was found to be tachycardic with a heart rate of 126 beats per minute and tachypneic with a respiratory rate of 55 breaths per minute. Within an hour after admission, her vitals spontaneously stabilized to be within normal limits. She was oriented to person and place, but not to time. She appeared disheveled and her behavior was belligerent and uncooperative. Affect was labile and irritable. Her speech was disorganized and rapid; the thought process was paranoid and easily distractible, and insight and judgment were poor.

On physical examination, there was no tenderness to palpation of the chest and neck and no palpable crepitus. All lung fields were clear to auscultation bilaterally; cardiac examination revealed a regular rate and rhythm without any murmurs, and the abdomen was soft and non-tender without any distension or guarding.

**Figure 1:**
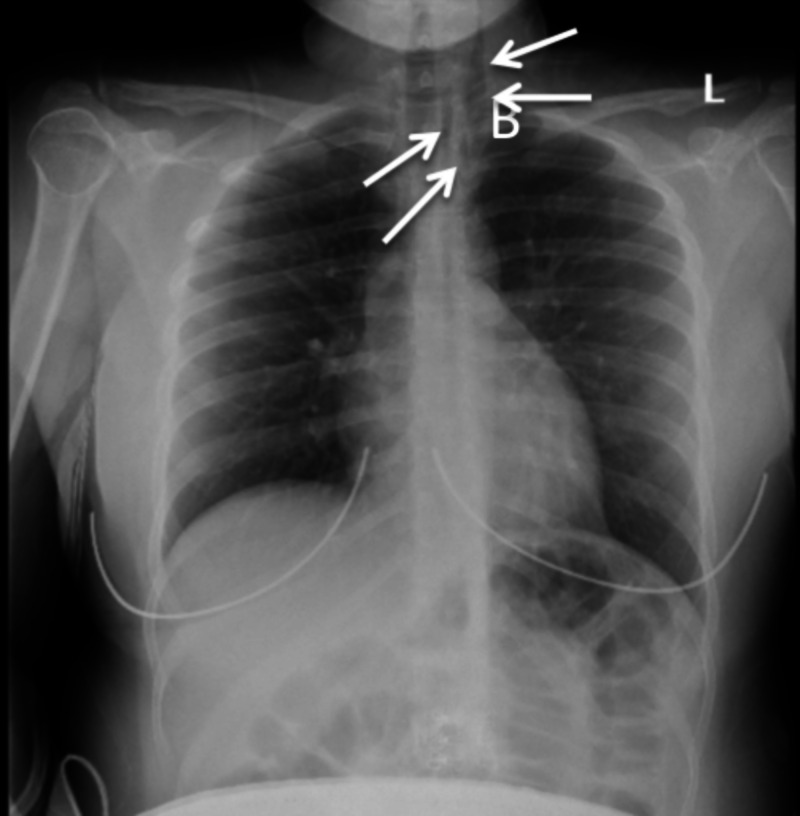
Upright chest X-ray (AP view) on hospital day one The image shows spontaneous pneumomediastinum at the base of the left neck (arrows) AP: anteroposterior

**Figure 2:**
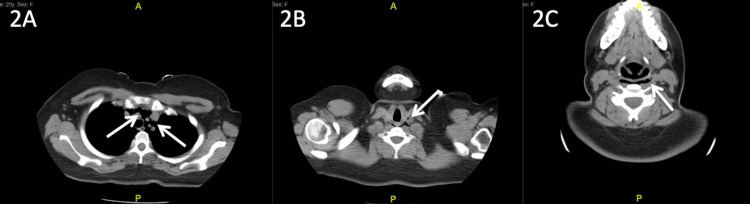
Non-contrast CT of the chest and neck (soft tissue window) on hospital day one Arrows point at subcutaneous air that can be seen in the superior mediastinum (2A), along the carotid sheath (2B), and retropharyngeal space (2C) CT: computed tomography

Initial labs were significant for leukocytosis of 13.6 K/mm^3^, low hemoglobin of 11.9 gm/dL with a mean corpuscular volume (MCV) of 85.8 fL, and low potassium of 3.0 mmol/L. A urine drug screen was positive for cannabinoids. An arterial blood gas test showed no significant acid-base abnormalities. Chest X-ray showed subcutaneous air along the base of the left neck (Figure 1), and subsequent non-contrast chest and neck CT scans showed air in the upper chest extending into the upper mediastinum, retropharyngeal soft tissue, and along the left common carotid (Figure 2). Notably, there was no evidence of esophageal rupture or underlying lung pathology.

The patient was initially admitted to the intensive care unit and closely monitored. She was treated with supportive care and NPO status. Empiric piperacillin/tazobactam was started due to initial concern for possible esophageal rupture. The patient was also evaluated and treated by the psychiatric service.

**Figure 3:**
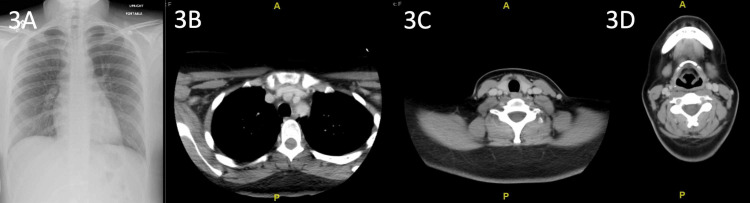
Repeat upright chest X-rays (AP view) and non-contrast CT of the chest and neck (soft tissue window) on the final hospital day The images show the resolution of pneumomediastinum in all previously noticed areas. There is no subcutaneous area present in the base of the left neck (3A), superior mediastinum (3B), along the carotid sheath (3C), or retropharyngeal space (3D) AP: anteroposterior; CT: computed tomography

The following day, the patient’s agitation and hallucinations resolved. Repeat chest X-ray showed improvement of her pneumomediastinum. An initial gastrografin study was inconclusive due to poor patient cooperation with the procedure. Conservative management was continued while serial chest X-rays were performed over the next three days. A chest X-ray and neck CT on hospital day four showed resolution of the pneumomediastinum (Figure 3). A repeat gastrografin study was successful and revealed no significant esophageal pathology. Noticeably, our patient remained asymptomatic throughout her hospital course, and serial physical examinations did not reveal any crepitus in the neck or chest or Hamman’s sign on auscultation.

Upon the resolution of the pneumomediastinum, our patient was subsequently transferred to the Behavioral Health Unit to continue treatment for her psychiatric condition. Her final diagnoses were an acute episode of psychosis secondary to polysubstance abuse and asymptomatic pneumomediastinum. She was discharged home after completing psychiatric treatment.

## Discussion

Our patient was a young woman who presented with a clinical picture of acute psychosis secondary to substance abuse. Chest X-ray revealed subcutaneous air along the base of the left neck, confirmed by CT of the neck and chest demonstrating extension of the subcutaneous air into the retropharyngeal soft tissue and the upper mediastinum. When pneumomediastinum is identified, the first critical step is to consider and rule out Boerhaave syndrome.

Boerhaave syndrome, or an effort rupture of the esophagus, is defined as the complete rupture of the esophagus caused by a sudden increase in intra-esophageal pressure; it is associated with mortality rates of up to 40% [11]. It is classically associated with severe vomiting and is most common in patients with a history of alcohol abuse. The classical presentation is a triad of vomiting, retrosternal chest pain, and subcutaneous emphysema, known as Mackler’s triad. However, the clinical presentation may vary based on the degree and location of rupture. Therefore, imaging plays an essential role in the diagnosis. The gold standard test of choice is a contrast esophagogram with water-soluble contrast gastrografin dye. Barium dye should be avoided as it can lead to further irritation of the mediastinal tissue in cases of true esophageal rupture. CT scan is a reliable alternative that has a higher sensitivity and allows for better visualization of involved organs. Due to our patient’s lack of symptoms, along with a negative esophagogram, Boerhaave syndrome was effectively ruled out. We then turned to consider the differential for pneumomediastinum. When doing so, it is helpful to distinguish between primary and secondary etiologies, which are outlined below.

Primary spontaneous pneumomediastinum 

Primary spontaneous pneumomediastinum (SPM) is defined as the presence of free air in the mediastinum without any underlying pathology. Common triggers include sports activities and heavy exertion, stool straining, labor, vomiting, and the Valsalva maneuver.

Secondary pneumomediastinum

Secondary etiologies can be further split into iatrogenic and non-iatrogenic. Iatrogenic causes include endoscopic procedures, intubation, pleural cavity instrumentation, and central venous access. Non-iatrogenic causes can either be traumatic (blunt or penetrating injuries), or non-traumatic [malignancy, esophageal rupture, pneumothorax, bronchiectasis, underlying lung conditions such as asthma and chronic obstructive pulmonary disease (COPD), performing pulmonary function tests (PFTs), and illicit drugs]. In cases secondary to drugs, it is believed that direct toxic effects on the lung parenchyma predispose the patient to pneumomediastinum. The correlation is strongest with cocaine, but associations have been reported with other recreational drugs such as marijuana and methamphetamines as well [12,13,14]. Marijuana use in young adults has also been shown to precipitate acute episodes of psychosis and worsen underlying psychosis [15].

Asymptomatic pneumomediastinum

Regardless of etiology, the description of asymptomatic pneumomediastinum is exceedingly rare in the literature. Cases that have described asymptomatic presentations have attributed them to causes such as dehydration and rhabdomyolysis, wisdom tooth extraction, thoracic epidural block, and isolated orofacial trauma [8,9,10]. It is possible, however, that many asymptomatic cases are simply not diagnosed as they do not seek medical attention.

The final diagnosis in our case was asymptomatic spontaneous pneumomediastinum, although there was an ongoing discussion about whether this was secondary to the patient's history of vomiting and illicit drug use. Typically, in the setting of marijuana or cocaine abuse, patients present with chest pain, dyspnea, throat discomfort, and/or subcutaneous emphysema. Our patient displayed no symptoms besides her psychosis, favoring a spontaneous etiology. It is important to note that our patient was initially tachycardic and tachypneic on presentation, which could have been symptoms of pneumomediastinum. However, these symptoms are not specific and could be due to her stress or recent substance use. In addition, her vitals quickly stabilized after presentation. This highlights the importance of objective imaging in the diagnosis and subsequent management of asymptomatic pneumomediastinum.

The management of spontaneous pneumomediastinum is typically conservative. Cornerstones of therapy include adequate rest and hydration, supplemental oxygen, and analgesics. Supplemental medications can include cough suppressants to help regulate intrathoracic pressure and anxiolytics as needed [2]. Smoking cessation is indicated in cases with underlying COPD. Most cases resolve within a few days, and serial imaging can be performed to confirm resolution. Since recurrence is not commonly observed, long-term follow-up is usually discouraged unless otherwise indicated [16].

## Conclusions

Primary spontaneous pneumomediastinum is a relatively rare diagnosis that predominantly affects young males. Symptoms include chest and neck pain, dyspnea, and subcutaneous emphysema, while asymptomatic cases are exceedingly rare. Diagnostic workup should include chest X-ray and confirmatory CT with contrast to help rule out more serious etiologies of pneumomediastinum and properly characterize the spread of air in the mediastinal space. Management is generally supportive and consists of rest and hydration, oxygen therapy, and analgesics. Patients can typically be discharged within a few days.
